# Telerehabilitation versus traditional centre-based pulmonary rehabilitation for people with chronic respiratory disease: protocol for a randomised controlled trial

**DOI:** 10.1186/s12890-018-0646-0

**Published:** 2018-05-15

**Authors:** Narelle S. Cox, Christine F. McDonald, Jennifer A. Alison, Ajay Mahal, Richard Wootton, Catherine J. Hill, Janet Bondarenko, Heather Macdonald, Paul O’Halloran, Paolo Zanaboni, Ken Clarke, Deidre Rennick, Kaye Borgelt, Angela T. Burge, Aroub Lahham, Bruna Wageck, Hayley Crute, Pawel Czupryn, Amanda Nichols, Anne E. Holland

**Affiliations:** 1 Discipline of Physiotherapy, La Trobe University and Institute for Breathing and Sleep, La Trobe University, Melbourne, VIC Australia; 2 Department of Respiratory Medicine Austin Health; Institute for Breathing and Sleep and University of Melbourne, Austin Health, Heidelberg, VIC Australia; 3 Discipline of Physiotherapy, University of Sydney and Sydney Local Health District, University of Sydney, Lidcombe, NSW Australia; 4 The Nossal Institute for Global Health, The University of Melbourne, Melbourne, VIC Australia; 5 Norwegian Center for E-health Research, University Hospital of North Norway, Tromsø, Norway; 6 Physiotherapy Department Austin Health and Institute for Breathing and Sleep, Austin Health, Heidelberg, VIC Australia; 7 Physiotherapy Department, Alfred Health, Prahran, VIC Australia; 8 Wimmera Health Care Group, Horsham, VIC Australia; 9 School of Psychology and Public Health, La Trobe University, Melbourne, VIC Australia; 10 Melbourne Networked Society Institute, University of Melbourne, Melbourne, VIC Australia; 11 West Wimmera Health Service, Nhill, VIC Australia; 12 Discipline of Physiotherapy, La Trobe University; Department of Physiotherapy, Alfred Health and Institute for Breathing and Sleep, La Trobe University, Melbourne, VIC Australia; 13 Discipline of Physiotherapy, La Trobe University, Melbourne, VIC Australia; 14 Monash Health, Clayton, VIC Australia

**Keywords:** Chronic obstructive pulmonary disease, Pulmonary rehabilitation, Telerehabilitation, Telehealth, Exercise, Respiratory disease, Interstitial lung disease, Bronchiectasis, Asthma

## Abstract

**Background:**

Pulmonary rehabilitation is an effective therapeutic intervention for people with chronic respiratory disease. However, fewer than 5% of eligible individuals receive pulmonary rehabilitation on an annual basis, largely due to limited availability of services and difficulties associated with travel and transport. The Rehabilitation Exercise At Home (REAcH) study is an assessor-blinded, multi-centre, randomised controlled equivalence trial designed to compare the efficacy of home-based telerehabilitation and traditional centre-based pulmonary rehabilitation in people with chronic respiratory disease.

**Methods:**

Participants will undertake an 8-week group-based pulmonary rehabilitation program of twice-weekly supervised exercise training, either in-person at a centre-based pulmonary rehabilitation program or remotely from their home via the Internet. Supervised exercise training sessions will include 30 min of aerobic exercise (cycle and/or walking training). Individualised education and self-management training will be delivered. All participants will be prescribed a home exercise program of walking and strengthening activities.

Outcomes will be assessed by a blinded assessor at baseline, after completion of the intervention, and 12-months post intervention. The primary outcome is change in dyspnea score as measured by the Chronic Respiratory Questionnaire – dyspnea domain (CRQ-D). Secondary outcomes will evaluate the efficacy of telerehabilitation on 6-min walk distance, endurance cycle time during a constant work rate test, physical activity and quality of life. Adherence to pulmonary rehabilitation between the two models will be compared. A full economic analysis from a societal perspective will be undertaken to determine the cost-effectiveness of telerehabilitation compared to centre-based pulmonary rehabilitation.

**Discussion:**

Alternative models of pulmonary rehabilitation are required to improve both equity of access and patient-related outcomes. This trial will establish whether telerehabilitation can achieve equivalent improvement in outcomes compared to traditional centre-based pulmonary rehabilitation. If efficacious and cost-effective, the proposed telerehabilitation model is designed to be rapidly deployed into clinical practice.

**Trial registration:**

Clinical trial registered with the Australian and New Zealand Clinical Trials Register at (ACTRN12616000360415). Registered 21 March 2016.

## Background

Chronic respiratory diseases, including chronic obstructive pulmonary disease (COPD), interstitial lung diseases (ILD), bronchiectasis and chronic asthma, contribute 7% to the global burden of disease [1]. Chronic respiratory diseases are the third leading cause of death worldwide, and account for 10% of all disability adjusted life years lost due to disability alone [2]. This level of disability is second only to that of cardiovascular disease, including stroke [2]. People with chronic respiratory disease experience repeated need for hospitalisation, reduced quality of life and life expectancy, poor exercise tolerance and physical functioning, and increased incidence of anxiety and depression [3]**.**

Pulmonary rehabilitation is a proven, effective strategy to achieve clinically important gains in exercise and functional capacity, symptoms and quality of life [4] across a variety of chronic respiratory diseases, including COPD [5], bronchiectasis [6], ILD [7] and asthma [8]. Participation in pulmonary rehabilitation also reduces hospitalisation due to acute exacerbations of respiratory disease [9] as well as overall healthcare utilisation [10]. Pulmonary rehabilitation is a recommended treatment strategy for individuals with a chronic respiratory disease in clinical guidelines across the world [4].

Despite compelling evidence for the benefit of pulmonary rehabilitation, only a very small percentage of eligible people ever attend a program [11]. There are well established barriers to uptake and participation in traditional centre-based pulmonary rehabilitation programs, both in the hospital and in the community, relating to referral practices, travel, transport, disability and lack of program staffing [12, 13]. Such barriers disproportionately compromise access to programs for patients in rural and regional locations [14]. In light of such obstacles, alternative modes of delivering pulmonary rehabilitation, in addition to traditional centre-based programs, are required to improve both equity of access and patient-related outcomes for people with chronic respiratory diseases and have been identified as a research priority [15].

Home-based models of pulmonary rehabilitation have been proposed to increase the availability and accessibility of pulmonary rehabilitation services to patients [16–20]. Recent work has demonstrated that home-based pulmonary rehabilitation achieves equivalent clinical outcomes to centre-based pulmonary rehabilitation [20]. However, a disadvantage of such programs is the lack of supervised exercise training. Advances in Internet technology and accessibility have made it possible for people to receive specialist medical care and therapeutic interventions straight to their home. Telerehabilitation is the use of information and communication technologies to provide clinical rehabilitation services from a distance [21]. Using the Internet, rehabilitation can be delivered directly to the patient’s location, regardless of physical proximity to a rehabilitation centre. Whilst telerehabilitation technology has existed for many years, the clinical efficacy of this model is not clear.

Preliminary studies have described the use of telerehabilitation in COPD, using a variety of program models. These studies suggest that telerehabilitation in COPD is safe, with no adverse events reported [22–28]. However, most existing trials have a number of important limitations, including: the requirement for participants to attend a health facility in order to access the telerehabilition service [22]; the use of bespoke, proprietary or poorly defined equipment to deliver telerehabilitation and monitor vital signs [23–25, 27]; failing to include supervised exercise training in the telerehabilitation model [23, 24, 27], and limiting the scope of application to individuals with COPD. These factors limit the clinical utility of previous telerehabilitation programs through restricted access [12], and omission of an essential component of pulmonary rehabilitation [4]. We have previously demonstrated the feasibility of a telerehabilitation model that delivers all the essential components of pulmonary rehabilitation into the home of people with COPD [28]. By using readily available equipment such as an exercise bike and a tablet computer it is possible for people to undertake a supervised exercise training program in their own home. However, to date, a comparison of the outcomes and costs of telerehabilitation to centre-based pulmonary rehabilitation has only been undertaken in the maintenance period post-rehabilitation [29]. A telerehabilitation model that allows all the essential components of pulmonary rehabilitation, specifically supervised exercise training and self-management education, to be delivered at home, using readily available equipment, with proven clinical outcomes and comparable costs, has the potential to dramatically change the uptake and accessibility of pulmonary rehabilitation for all patients with a chronic respiratory disease.

Analysing the cost of telerehabilitation is critical to determine the economic viability of implementing such a model into clinical practice. To date, there is a lack of evidence to support the cost-effectiveness of telerehabilitation, despite apparent clinical benefits in a range of health conditions [21]. A comprehensive assessment of the economic value of telerehabilitation needs to include both costs to the healthcare system, including the initial costs of equipment and its transport [30], together with costs to the patient [31]. Telerehabilitation has the potential to overcome many known barriers to pulmonary rehabilitation participation and, if cost-effective, could be a relevant treatment alternative across all chronic respiratory diseases where rehabilitation is an accepted therapeutic intervention.

This paper describes the protocol for the Rehabilitation Exercise At Home (REAcH) trial – a study of telerehabilitation in chronic respiratory disease. The aims of the study are to compare the: 1) clinical outcomes of telerehabilitation and traditional centre-based pulmonary rehabilitation for people with a chronic respiratory disease; 2) costs of telerehabilitation and centre-based pulmonary rehabilitation. We hypothesise that the clinical effects on symptoms, exercise capacity, and health-related quality of life (HRQoL) will be equivalent between pulmonary rehabilitation models; that the proportion of participants who complete pulmonary rehabilitation will be greater in the telerehabilitation group; and, that telerehabilitation, delivered using our low-cost model, will provide a cost-effective alternative to centre-based pulmonary rehabilitation (from a societal perspective) for people with chronic respiratory disease.

## Methods

### Design

A randomised, controlled, assessor-blinded equivalence trial with an embedded economic evaluation will be conducted at two centres in metropolitan Melbourne (Alfred Health and Austin Health), and one regional centre (Wimmera Health Care Group, Horsham), Australia. Both regional and metropolitan sites have been included to increase the external validity of our model. The study was approved by the Human Research Ethics Committee at Alfred Health for all sites, and local governance approvals obtained from all participating sites (Austin Health, Wimmera Health Care Group and West Wimmera Health Service). The trial was registered at anzctr.org.au (ACTRN12616000360415) on March 21, 2016.

### Participants

Potential participants will be referred to pulmonary rehabilitation at the established centre-based programs of the participating sites, or those individuals admitted to hospital with an exacerbation of their respiratory disease. To be eligible for inclusion participants will: 1) have a primary diagnosis of a chronic lung disease; 2) be aged ≥40 years; and 3) be able to read and speak English. Potential participants will be excluded if they have: 1) a primary diagnosis of pulmonary hypertension or lung cancer, as rehabilitation is not yet a well established treatment for these diagnoses; 2) attended pulmonary rehabilitation within the previous 18 months and had no hospitalisations for a respiratory cause since rehabilitation completion, as these patients may not achieve further benefits; 3) oxygen desaturation resulting in cessation of cardiopulmonary exercise testing (e.g. SpO_2_ < 80% during exercise on cycle ergometer), to ensure safety of unsupervised training; 4) unstable or brittle asthma with a hospital admission or emergency department presentation within the preceding 3 months, to ensure safety of unsupervised training; 5) co-morbidities which preclude exercise training, such as neurological or musculoskeletal impairment; or 6) are unable to follow verbal instructions, suffer from cognitive impairment, or have language difficulties. Eligible participants will be provided with written and verbal information about the study from a clinician and/or a researcher. All participants will provide written consent. Trial participation will have no effect on routine management of their respiratory condition or any other healthcare requirements.

### Recruitment and randomisation

Participants will be randomly allocated (1:1) to traditional centre-based pulmonary rehabilitation or telerehabilitation. A computer-generated, block randomisation scheme will be used with stratification for (i) recruitment in stable state vs post hospitalisation for respiratory exacerbation; (ii) site of recruitment; and (iii) diagnosis of ILD vs other diagnoses. Sequence generation will be performed by an individual who is independent of the research team and randomisation will occur using an online database. The randomisation sequence will be concealed from investigators. Participants will be allocated to groups after completion of baseline assessment. Given the nature of the intervention (exercise training) participants will not be blinded to the intervention, however all outcomes will be measured by an independent assessor blind to group allocation. The flow of participants through the study will be reported according to the recommendations of the Consolidated Standard of Reporting Trials (CONSORT) [32].

### Interventions

#### Centre-based pulmonary rehabilitation

Participants will undergo a standard outpatient pulmonary rehabilitation program at the centre where they were recruited, according to Australian best-practice guidelines [33]. This involves 8 weeks of twice-weekly supervised sessions in groups of 8–12 participants, supervised by a health professional experienced in the delivery of pulmonary rehabilitation (eg. physiotherapist, exercise physiologist) and appropriately qualified assistants (eg. allied health assistant, nurse). Participants will undertake at least 30 min of lower limb aerobic training each session, which may be completed in shorter intervals if continuous training is limited by symptoms (eg. 3 × 10 minutes or 2 × 15 minutes). A combination of cycling and walking will be used, with the initial walking exercise intensity set at 70–80% of the speed walked on a 6-min walk test and cycle ergometer intensity set at the work rate equivalent to 60% of the peak oxygen uptake (VO_2_) on a cardiopulmonary exercise test (CPET) [34]. Intensity of both cycle and walking training will be progressed each week with the aim of maintaining Borg dyspnoea scores of 3–4.Cycle training will be progressed by 5–10% of the initial workload [35] and walking speed by 0.25 or 0.5 km/hour depending on initial training speed (< 3 km/hour or ≥ 3 km/hour respectively). Resistance training will utilise functional activities such as sit-to-stand from a chair and upper limb weights. Initial weights will be prescribed as tolerated, to achieve 8–12 repetitions for 3 sets of each exercise. Once the participant achieves 3 sets of 12 repetitions then the weight will be increased. Participants will be encouraged to perform an additional 3 unsupervised sessions each week, which will be documented in a home diary that is reviewed weekly by the supervising physiotherapist. A record of sessions attended by each participant will be maintained. Exercise training will be standardised across all sites with the use of a protocol for prescription and progression.

#### Telerehabilitation

Remotely supervised telerehabilitation at home will be conducted twice a week for 8 weeks, in groups of 4–6 participants at a time, supervised by an experienced physiotherapist. Exercise training will comprise 30 min of lower limb aerobic training and individualised strength training exercises. Participants will undertake a total of 30 min of cycle training, in two or more bouts, in each telerehabilitation session. Cycle ergometer intensity for telerehabilitation participants will be set with the same parameters as for centre-based participants, that is, at the work rate equivalent to 60% of the peak oxygen uptake (VO_2_) on a cardiopulmonary exercise test (CPET) [34]. Intensity of cycle training will be progressed each week by 5–10% of the initial workload as tolerated [35], based on patient symptoms. Resistance training for the arms and legs will utilise equipment readily available in the home environment (e.g. sit to stand from a standard height chair, bags of rice for upper limb weights). Resistance training will be initially prescribed as tolerated, to achieve 8–12 repetitions for 3 sets of each exercise. Similar to the centre-based group, telerehabilitation participants will also be encouraged to perform an additional 3 unsupervised sessions each week, which will be documented in a home diary that is reviewed weekly by the supervising physiotherapist.

The telerehabilitation set-up will be based on our successfully piloted model [28] using readily available equipment. It will comprise a step-through exercise bike to maximise safety (Bodyworkx A915); a tablet computer (iPad – for relative ease of use and limited need to negotiate menu functions) fixed to a stand for videoconferencing; and a pulse oximeter (Nonin Palmsat 2500A; Nonin Medical Inc., Plymouth, Minnesota, USA) to monitor SpO_2_ and heart rate during training and at rest. The oximeter will not use Bluetooth but will be positioned such that the display is visible to the supervising physiotherapist throughout the session. Videoconferencing will be via Zoom videoconferencing software (San Jose, California, USA) that enables all participants to see and speak to each other. To ensure safety and understanding of equipment operation and the exercise program, the initial training session and establishment of the home exercise program will occur during a home visit by a physiotherapist.

Fidelity of the exercise training intervention for both groups will be facilitated through regular staff training, audit of exercise prescription and progression, and assessment of participant engagement [36].

Disease-specific education and collaborative self-management training will be delivered to participants in both groups according to international guidelines [4]. To standardise provision of information, participants in both groups will receive self-management education resources from Lung Foundation Australia in the form of a printed book and brochure detailing the location of online resources [37]. These resources have been developed to enable people with chronic respiratory diseases to undertake the educational component of pulmonary rehabilitation at their convenience. Self-management education for all participants will include long-term exercise planning and, for patients with COPD or asthma, education regarding identifying and managing an acute exacerbation. Additional topics relating to self-care in chronic respiratory disease, such as airway clearance techniques and strategies for maintaining a healthy diet will also be addressed where participants identify a relevant health goal. After the 8-week rehabilitation program, all participants in both groups will be encouraged to continue with a regular exercise regimen. All participants will be offered the opportunity to join a supervised exercise maintenance program to promote ongoing exercise adherence, in line with national standards [33]. The proportion of each group that accept a referral and attend a program will be documented.

##### Outcome measures

Demographic details of age, gender, diagnosis, body mass index (BMI) and lung function will be collected at baseline. A maximal CPET using cycle ergometry with expired gas analysis will be completed as part of the baseline assessment to establish a work rate for an endurance cycle test (see secondary outcomes). Attendance rates at training sessions will be documented at the end of the intervention period and compared between the centre-based and telerehabilitation groups, with an a priori definition of pulmonary rehabilitation adherence (i.e. program completion) as undertaking a minimum of 70% of planned pulmonary rehabilitation sessions [38].

**Fig. 1:**
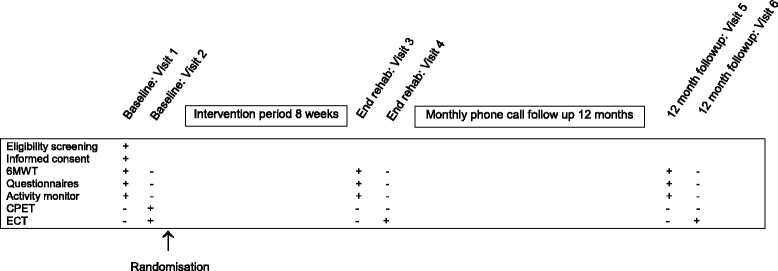
Participant schedule. Legend: + = completed; − = not completed; 6MWT = six minute walk test; CPET = cardiopulmonary exercise test; ECT = endurance cycle test

Participants will undertake assessment of clinical outcome measures at baseline, end intervention and after 12-months follow-up (see Fig. 1). The following measures will be recorded:

##### Primary outcome

The Chronic Respiratory Disease Questionnaire (CRQ) is a disease specific measure of HRQoL for people with chronic respiratory disease [39]. The CRQ scores the domains of dyspnea, fatigue, mastery and emotional function. The primary outcome will be change in CRQ dyspnea domain (CRQ-D) from baseline to end intervention. The CRQ-D is a valid measure of HRQoL and is responsive to change with pulmonary rehabilitation in people with chronic respiratory diseases including COPD [40], bronchiectasis [41] and ILD [42].

##### Secondary outcomes

Exercise capacity will be measured using both the 6-min walk distance (6MWD) and endurance cycle time. The 6MWD will be measured using the 6-min walk test which is a validated measure of functional exercise capacity [43], and is responsive to change following pulmonary rehabilitation in people with chronic respiratory diseases [44, 45]. Two tests will be completed at each assessment time-point according to standard procedures and the best distance used for analysis [43]. Endurance cycle time will be measured during a constant work rate exercise test, performed at 75% of the peak work rate on the baseline CPET. Endurance cycle time is highly responsive to therapeutic interventions in people with chronic respiratory disease [46, 47].

The CRQ domains of fatigue, mastery and emotional function, reflecting HRQoL, will be reported as secondary outcomes. The SF-36v2, a validated generic quality of life measure will be used to derive a preference-based measure of utility, the SF-6D, for economic analyses [48].

Self-efficacy will be measured using the Pulmonary Rehabilitation Adapted Index of Self Efficacy (PRAISE) tool, a reliable and sensitive measure of self-efficacy for patients attending pulmonary rehabilitation [49].

Dyspnea will be measured using the Modified Medical Research Council scale [50].

Anxiety and depression will be evaluated using the Hospital Anxiety and Depression Scale [51]. Anxiety and depression are common comorbidities in people with chronic respiratory disease and can be ameliorated with pulmonary rehabilitation [4].

Physical activity levels will be measured objectively using a wrist-worn activity monitor over seven days (GeneActiv; ActivInsights Ltd., Kimbolton, Cambridgeshire, United Kingdom). The GeneActiv is a tri-axial accelerometer that is validated for the assessment of free-living activity in healthy people [52] and has been used to assess physical activity in people with chronic respiratory disease [53]. Data will be collected and stored as raw acceleration in g units (m/s^2^) for offline analysis. Time (minutes) per day spent in sedentary, light, moderate and vigorous physical activity will be reported.

### Economic evaluation

Economic evaluation will take a societal perspective [54]. Economic evaluation will incorporate costs for all stakeholders involved, including participants’ out-of-pocket costs associated with health care services, and the costs of health services, including intervention costs. To facilitate documentation of personal health care costs, participants will be contacted by telephone every month during the 12-month follow-up period to document self-reported health care utilisation and to encourage completion of the monthly diary. Individual-level data for outcomes of Quality-Adjusted Life Years (QALYs), CRQ-D scores and costs will be compared to assess the incremental impact of telerehabilitation compared to traditional centre-based pulmonary rehabilitation. To compare differences in costs with differences in QALYs the SF6D score, which determines an individual’s self-valuation of health state at a specific time-point and is generated from the SF36-v2, will be converted into QALYs [55]. In addition, a separate assessment of cost effectiveness will also be undertaken by comparing the change in CRQ-D scores from baseline to end rehabilitation to the incremental costs of the intervention.

Direct costs of telerehabilitation may be higher than those for centre-based pulmonary rehabilitation, due to capital expenditure for equipment, but these may be accompanied by savings in the form of averted expenditure on health service use. Thus we will also calculate the (internal) rate of monetary return from investing in the intervention, which compares the difference in costs of health service use associated with telerehabilitation and centre-based rehabilitation to initial outlay for infrastructure [30]. For this purpose, any savings from lowering health system costs will be assessed over a time horizon of 12-months [30]. Sensitivity analyses will capture variations in cost of establishing telerehabilitation in rural versus urban settings in Australia.

### Statistical analysis

#### Sample size

If there is truly no difference in the change in the dyspnea domain of the CRQ between telerehabilitation or traditional centre-based pulmonary rehabilitation at the end of the 8 week intervention, then 128 participants (64 in each group) are required to be 80% sure that the 95% confidence interval will exclude a difference in means of 2.5 points or more. This is the minimal important difference (MID) for the CRQ-D [56] and assumes a standard deviation of the change in CRQ-D of 4.8 points [57]. A total of 142 participants will be randomised to allow for 10% attrition. It is anticipated that recruitment will be evenly distributed across sites.

This sample size will also give sufficient power for secondary outcomes:

- *6-min walk distance (6MWD)*: if there is truly no difference in 6MWD at the completion of 8-weeks of centre-based pulmonary rehabilitation or telerehabilitation, a total of 108 participants will be required to ensure that the 95% confidence interval excludes a difference in group means of 30 m or more [43], assuming a standard deviation of change in 6MWD of 53 m [58].
- *Endurance Cycle Time****:*** if there is truly no difference between telerehabilitation and centre-based pulmonary rehabilitation in change in constant work rate endurance time on cycle ergometer, then 52 participants are required to be 80% sure that the 95% confidence interval will exclude a difference in means of 150 s or more. This is the MID for endurance time for patients with COPD [59] and assumes a standard deviation of 184 s [59].
- *Pulmonary rehabilitation adherence (program completion):* data from our centres indicate that only 65% people who are referred to pulmonary rehabilitation take up the referral and complete the program. Previous studies have documented completion rates for telerehabilitation programs of over 90% [23, 60]. Using a conservative estimate of 85% completion, 128 patients will be required to detect a difference in completion rates between telerehabilitation and traditional pulmonary rehabilitation.

#### Analysis

Continuous variables will be analysed by fitting linear mixed models, controlling for recruitment centre and baseline values as required. The proportion of participants who attend for at least 70% of the program will be compared between groups using a chi-squared test and the relative risk of non-completion will be determined. All data will be analysed by intention-to-treat. A per-protocol analysis will also be conducted to reduce the risk of Type 1 error, as recommended in the CONSORT Extension for reporting of non-inferiority and equivalence trials [61]. Alpha will be set at 0.05.

#### Data integrity and management

Data will be stored on a purpose-built online database (www.adeptrs.com), with encryption, password protection and restricted access. No identifying information will be stored in the online database.

#### Withdrawal

A participant will be considered to have withdrawn from the study when consent is revoked. If this occurs, no further assessments will be performed. Participants will be informed that data collected up to the time of withdrawal will form part of the study results unless permission is expressly declined. Withdrawn participants will not be replaced. Protocol violations will not constitute grounds for withdrawal. Study withdrawal will not have any impact on care provided by any of the participating sites.

#### Monitoring

The trial will be monitored by an independent Data Safety Monitoring Board (DSMB) comprising a respiratory physician and two clinical research physiotherapists, with consultation with a statistician as required. The DSMB will review data relating to the primary outcome (CRQ-D) as well as 6MWD, endurance cycle time and Hospital Anxiety and Depression Scale scores. Data will be presented to the DSMB in a blinded fashion. The DSMB will initially review data at a time 6 months from the commencement of recruitment. Any serious adverse events will be notified immediately to the overseeing ethics committee (Alfred Health) and the relevant site governance committee, as well as to the DSMB. If there are concerns about the safety of participants, the DSMB will make a recommendation to the trial steering committee about continuing, stopping, or modifying the trial.

## Discussion

Pulmonary rehabilitation is a key recommended component of the non-pharmacological management of individuals with chronic respiratory disease [4], yet limited access to programs prevents the widespread application of its benefits. This study will compare both the clinical- and cost-effectiveness of delivering pulmonary rehabilitation via telerehabilitation, using readily available consumer devices and equipment, to a traditional centre-based program.

The telerehabilitation model under investigation directly addresses access barriers for both individual patients and the health system. By delivering pulmonary rehabilitation directly into the homes of people with chronic respiratory disease issues of transport, travel, their associated costs and weather could be negated [13, 62]. By improving ease of access, more individuals may have the opportunity to develop an informed and positive perception of the benefits of pulmonary rehabilitation. Reduced healthcare utilisation has also been reported following completion of pulmonary rehabilitation [63], and thus implementation of telerehabilitation programs has the potential to positively impact on healthcare expenditure.

Unlike previous studies [23–25, 27] the telerehabilitation model under investigation uses equipment that is familiar to clinicians and patients, requires little technical support, is scalable, and has the potential to be easily implemented into clinical practice. Telerehabilitation can be delivered any time and from any place, further extending access to patients who live away from metropolitan centres. By providing supervised exercise training in this way, pulmonary rehabilitation could be accessed in locations where specialist services would ordinarily be unavailable [64]. The remote supervision of exercise training in this telerehabilitation model is a key feature frequently unavailable in models of pulmonary rehabilitation not based in a healthcare setting. In a recent study of home-based pulmonary rehabilitation, 80% of individuals who did not wish to take part in the study declined because they wanted to attend the supervised pulmonary rehabilitation group [20]. By providing supervised exercise remotely, individuals who would otherwise be reluctant to exercise without the support of a healthcare professional may be more inclined to participate.

The purpose of this study conforms with policy statements to encourage the investigation of alternative models of pulmonary rehabilitation delivery in order to enhance implementation, access and delivery of pulmonary rehabilitation [15]. If this study demonstrates that telerehabilitation has equivalent clinical outcomes to centre-based pulmonary rehabilitation, and is cost-effective, this model has the potential to significantly increase service availability and accessibility through increased options for pulmonary rehabilitation delivery.

### Trial status

Recruitment commenced in August 2016 and is continuing.

## Abbreviations

6MWD: Six minute walk distance
BMI: Body mass index
COPD: Chronic obstructive pulmonary disease
CPET: Cardiopulmonary exercise test
CRQ: Chronic respiratory disease questionnaire
CRQ-D: Chronic respiratory disease questionnaire – dyspnea domain
DSMB: Data safety monitoring board
HRQoL: Health related quality of life
ILD: Interstitial lung disease
km/hour: kilometres per hour
MID: Minimal important difference
QALYs: Quality adjusted life years
SF-36v2: 36 item short-form health survey version 2
VO_2_: Oxygen uptake
